# Iliotibial band syndrome rehabilitation in female runners: a pilot randomized study

**DOI:** 10.1186/s13018-020-01713-7

**Published:** 2020-05-24

**Authors:** Janine McKay, Nicola Maffulli, Rocco Aicale, Jack Taunton

**Affiliations:** 1 Emirates Integra Medical and Surgery Centre, Dubai, UAE; 2 Department of Musculoskeletal Disorders, School of Medicine and Surgery, University of Salerno, Salerno, Italy; 3 Clinica Ortopedica, Ospedale San Giovanni di Dio e Ruggi D’Aragona, 84131 Salerno, Italy; 4 Queen Mary University of London, Barts and the London School of Medicine and Dentistry, Centre for Sports and Exercise Medicine, Mile End Hospital, 275 Bancroft Road, London, E1 4DG England; 5 School of Pharmacy and Bioengineering, Keele University School of Medicine, Thornburrow Drive, Stoke on Trent, England; 6 Allan McGavin Sports Medicine Centre, Vancouver, BC Canada; 7 Fortius Institute, Burnaby, BC Canada

**Keywords:** Hip strengthening exercise, Stretching, Pain reduction, Female runners, Iliotibial band syndrome, Rehabilitation

## Abstract

**Background:**

Iliotibial band syndrome (ITBS) carries marked morbidity in runners. Its management is not standardized and lacks evidence base. We evaluated the effectiveness of three different exercises programs in reducing ITBS symptoms.

**Methods:**

Patients were divided into three equal treatment groups: ITB stretching (group A), conventional exercise (group B), and experimental hip strengthening exercise (group C). Numeric pain rating scale (NPRS; every week), lower extremity functional scale (LEFS; every 2 weeks), dynamometer (DN; weeks 0, 2, 4, 6, 8), single-limb mini squat (SLMS; week 0, 8), and Y-balance test™ (YBT), between and within group’s differences were evaluated using ANOVA model.

**Results:**

Twenty-four female runners (age 19–45 years) were included into one of three groups (A, B, and C). Statistical significance (*p* < 0.05) within group C was observed for composite YBT and DN for injured and non-injured leg, the YBT (injured leg for the posterior medial), LEFS, NPRS, and the SLMS. Statistical significance (*p* < 0.05) was found between group A and group C. The stretching group exhibited statistically significant (*p* < 0.05) YBT anterior reach for the injured/non-injured leg and the LEFS.

**Conclusion:**

There were no statistical differences between the three groups. The subjects who underwent experimental hip strengthening exercises consistently showed improvements in outcome measures, and never scored less than the other two groups.

**Trial registration:**

ClinicalTrials.gov identifier (NCT number): NCT0229615

## Background

Iliotibial band syndrome (ITBS) is one of the most common injuries among runners [1], with two different competing anatomic theories for ITBS, namely, compression and enthesopathy versus friction and impingement [2]. The incidence of ITBS has almost doubled from 1981 to 2000, raising from 4.3 to 8.4% among the population of patients presenting with running injuries [1, 3, 4]. In the early 1980s, the prevalence of ITBS in female and male runners was reported to be 34% and 65.4%, respectively [5]. At the beginning of this century, a retrospective case-control analysis of running injuries reported the prevalence in women and men to be 62% and 38%, respectively [1].

Some risk factors associated with the occurrence of ITBS include history of previous injuries, age (< 34 years), tight lateral fascial band (also known as the ITB), interval training, improper foot wear, running surface, high weekly mileage, lack of recovery, downhill running, leg length inequalities, increased angle of flexion of the knee at heel strike, and muscular weakness of knee extensors, knee flexors, and hip abductors [1, 6–9].

Treatment of ITBS may be undertaken using conservative or surgical methods, with surgery indicated in refractory cases. Surgical treatment includes excision or release of the affected distal portion of the ITB to loosen or lengthen it, or bursectomy [10, 11]. Surgical interventions should only be considered if long-term conservative management is ineffective. Non-surgical methods or conservative management are preferred and may include a combination of rest, activity modification, pain management, stretching, and strengthening. The conservative therapies used for the treatment of runners presenting with pain include the use of non-steroidal anti-inflammatory drugs (NSAIDs), corticosteroids, deep friction massages, phonophoresis, ice packs, ultrasound, and stretching. Although several studies have reported the effects of conservative therapies in reducing pain, current management options of ITBS have not been clearly established. To our knowledge, no randomized study to date has assessed the effects of individual conservative therapies [2, 12–16].

Patients with ITBS are recommended to take rest as the first step to recover from pain [12]. Thus, refraining from the activities which incite the pain followed by a gradual return is suggested to avoid recurrence of symptoms [12]. Addition of physical therapy to other forms of conservative treatments is essential to treat ITBS. Medications are only good to treat pain and inflammation. Treatment strategy should include physical therapies including stretching the ITB, and strengthening the hip abductors to recover completely from the injury [17].

A “progressive 3-phase model” exercise regimen has been found to be an effective rehabilitation program for the lower extremity [18]. Phase one includes seated hip strengthening exercises followed by phase two which includes continuation of the exercises in phase one but with increasing intensity in balance exercises, and phase three involves stopping of exercises and gradually returning of the patient to normal sport activities. The patients are assessed throughout their treatment and only progress to the next stage of more difficult activities if the milestone is met [19].

There is a lack of evidence on how lower extremity injuries specifically impact females and related biomechanics. Studies have not consistently distinguished between male and female runners, and have produced complicated predictors that apply in some but not all cases and have led to treatment regimens which are not always based on clinical evidence [2, 18, 20]. Further, there is lack of gender-specific studies assessing the efficacy of differentiated treatment. The present study aimed to assess the effectiveness of three different exercise regimens (stretching, conventional hip rehabilitation, and experimental exercises which involve progressive increase in complexity) in female runners with ITBS.

## Methods

This randomized study was conducted at Avita Health and Massage Centre and Optimal Performance Clinic for a period of 8 weeks between November 2014 and November 2015. The study was approved by the Clinical Research Ethics Board of the University of British Columbia (UBC-REB). All the eligible participants signed the UBC-REB approved consent form prior to their first assessment visit. ClinicalTrials.gov identifier (NCT number): NCT0229615.

Female distance runners aged 19–45 years, self-classified as recreational (running a minimum average of 15 running kilometers per week) runners with unilateral ITBS for at least 3 months were included.

Individuals with a history of previous knee trauma or knee surgery; other knee abnormalities including patellofemoral joint pain, popliteus tendinitis, lateral meniscal injury, degenerative joint disease, and lateral collateral ligament sprain to the affected side; and any previous treatment for ITBS were excluded.

The present study aimed to evaluate the relationship between hip strengthening and each of the following: pain associated with ITBS, YBT outcome, SLMS as a functional outcome, and LEFS, a self-reported questionnaire.

The study further examined the differences between groups assigned ITB stretching, conventional exercise, and experimental hip strengthening exercise; and their effects on the above outcome measures. Additionally, the difference of all three treatment groups (stretching, conventional exercise, and experimental exercise) on the injured versus non-injured leg was determined.

A licensed chiropractor determined the diagnosis of ITBS by medical history and physical examination. Lateral knee pain was reported to be the prominent symptom in all runners that worsened with running, especially downhill. Diagnosis was confirmed by the presence of local tenderness over the lateral epicondyle; reproducible pain with Nobel’s compression test; pain with flexion and extension of the knee while exerting pressure over the lateral femoral condyle with maximal pain at about 30° of knee flexion; and the absence of any other knee joint pathologies such as meniscal injury, arthritis, ligament injury, and popliteal tendonitis.

The study participants were divided into three treatment groups: a control group (group A), which underwent a programme of stretching, conventional exercise (group B), and experimental hip strengthening exercise (group C). Weekly progression of the exercises in group A and group B is presented in Table 1 and for group C is presented in Table 2.

**Table 1:** Weekly progression of exercise in stretching and conventional hip exercise group

Treatment groups and exercise regimen	Duration in weeks (s and repetitions per day)			
	0–2	2–4	4–6	6–8
Stretching: group A				
Trunk side bend ITB stretch	30 × 2	30 × 2	40 × 3	40 × 4
Trunk side bend with reach-ITB stretch	30 × 2	30 × 2	40 × 3	40 × 4
ITB stretch	30 × 2	30 × 2	40 × 3	40 × 4
ITB hip abductor stretch	30 × 2	30 × 2	40 × 3	40 × 4
Conventional hip exercise: group B				
Hip external rotation clamshells	10 × 2 medium T-band	10 × 3 light T-band	15 × 3	15 × 3 medium T-band
Side-lying hip abduction	10 × 2	10 × 3	15 × 3	20 × 3
Reverse internal rotation clamshell	10 × 2	10 × 3	15 × 3	20 × 3
Supine bridge	10 × 2 medium T-band	10 × 3 light T-band	15 × 3	15 × 3 medium T-band

Frequency of all exercises was 3 days/week

Abbreviation: *ITB* iliotibial band

**Table 2:** Weekly progression of exercise in experimental hip strengthening exercise group (group C)

Exercise regimen	Duration (s and reps × sets)
Exercise 1: Progression	
Modified side plank	Week 1: 30 × 2*
	Week 2: 40 × * (Each side)
Modified side plank with clamshell	Week 3: 10 reps x 2 Light T-band
	Week 4: 15 reps × 3 Light T-band
Side plank	Week 4–6: 15 s × 2* and 20 s × 3 (each side)
Side plank with hip abduction	Week 6–8: 10 reps × 2 and 10 reps × 3
Exercise 2: progression	
Side-lying hip abduction	Week 1: 10 × 2
	Week 2: 10 × 3
Lateral monster walk	Week 3: 15 × 2 Light T-band**
	Week 4: 15 × 3 Light T-band**
Monster walk with shoulder external rotation	Week 5: 15 × 2 Light T-band**
	Week 6: 15 × 3 Medium T-band**
Monster × walk	Week 7: 15 × 2 Light T-band**
	Week 8: 15 × 3 Medium T-band**
Exercise 3: progression	
Hip hikes	Week 1: 10 × 3***
	Week 2: 20 × 3***
Single leg squat	Week 3: 10 × 2***
	Week 4: 12 × 3***
TKE with T-band-hip abduction	Week 5: 12 × 2 Light T-band***
	Week 6: 12 × 3***
Skater-running man	Week 7: 15 × 2***
	Week 8: 15 × 3***
Exercise 4: progression	
Supine bridge with T-band progression	Week 1: 10 × 3 medium T-band
	Week 2: 15 × 3
Cook hip lift	Week 3: 10 × 2***
	Week 4: 10 × 3***
Glute bridge with single leg march	Week 5: 10 × 2***
	Week 6: 12 × 3***
Double leg hip thrust-bottom up	Week 7: 12 × 2
	Week 8: 15 × 3

Note: Frequency of all exercises was 3 days/week.

Abbreviation: *T-band* Theraband, *TKE* terminal knee extension

*Each side

**Each direction

***Each leg

The experimental exercises were designed by a kinesiologist and strength and conditioning coach, certified by the National Strength and Conditioning Association (NSCA). The exercises chosen for the experimental group were built upon previous research from side-lying exercises and were designed to progressively increase in complexity over 8 weeks. The complexity was increased by adding multi-jointed exercises, advancing from side-lying to standing positions, bilateral to unilateral and adding some upper extremity movements. Participants were also instructed to avoid involvement in any painful activities, until their pain was under control, and then gradual reintroduction of activity was recommended.

Gradual reintroduction of running was started once participants achieved pain free status, after 30 min of fast walk. The gradual reintroduction program was designed to slowly return subjects to running following injury. The gradual, progressive approach of this program was designed to give the body an opportunity to continue healing without causing further damage (Table 3).

**Table 3:** Gradual return-to-run program

Week	Day (alternating)	Walk (min)	Run (min)	Frequency of interval
1	Monday	4.5	0.5	6
	Wednesday	4.0	1.0	6
	Friday	3.5	1.5	6
2	Monday	3.0	2.0	6
	Wednesday	2.5	2.5	6
	Friday	2.0	3.0	6
4	Monday	1.5	3.5	6
	Wednesday	1.0	4.0	6
	Friday	0.5	4.5	6
5	Monday	0.0	30.0	1

^**2**^Note**:** total time was 30 min for all days

The NPRS documentation sheet for pain was given to all participants weekly. The NPRS is a universal scale for rating pain (0–10; where 0 is “no pain” and 10 is “worst possible pain”) for measuring the self-reported pain intensity in patients. The NPRS is short, easy to administer, and has been validated as a measure of pain intensity in populations with known pain [21, 22].

All subjects were required to complete the LEFS sheet at 2-week intervals. The LEFS tool is a commonly used reliable outcome measure for individuals with a broad spectrum of lower extremity injuries [23].

Hip abductor strength was tested at two-week intervals, at weeks 0, 2, 4, 6, and 8. The MicroFET2™ (Hoggan Health Inc., UT, USA), a wireless digital hand-held dynamometer (HHD), a load-cell recording device that displays a digital readout in pounds of force and length of time of muscle contraction, was used [24].

Bilateral SLMS was used to assess the quality of movement, which was measured at week 0 and week 8. The SLMS has been validated as a clinical test for assessing quality of movement by visual analysis [25].

The YBT™ is an instrumented version of components from the star excursion balance test (SEBT) and was developed to improve the repeatability of measurements and standardization for performance of the test. The YBT™ can be separated into lower and upper quarter. This study used the lower quarter (LQ) to evaluate the participants’ lower extremity and pelvis function, specific to the sport of running [26].

Variability was controlled between subjects by including age, gender, and leg of injury in the study model. Power analysis based on a t-test for each coefficient estimate of ANOVA model was performed. Pooled standard deviation (SD) was found to be 2.34% BWh. LEFS measures were controlled for, at week 0, as an intercept in the repeated measurements model. Significance level of 0.05 was considered statistically significant. One-sided alternative hypothesis was used for power analysis. All statistical procedures were performed using the R Project for statistical computing (R Core Team, 2015).

## Results

**Fig. 1:**
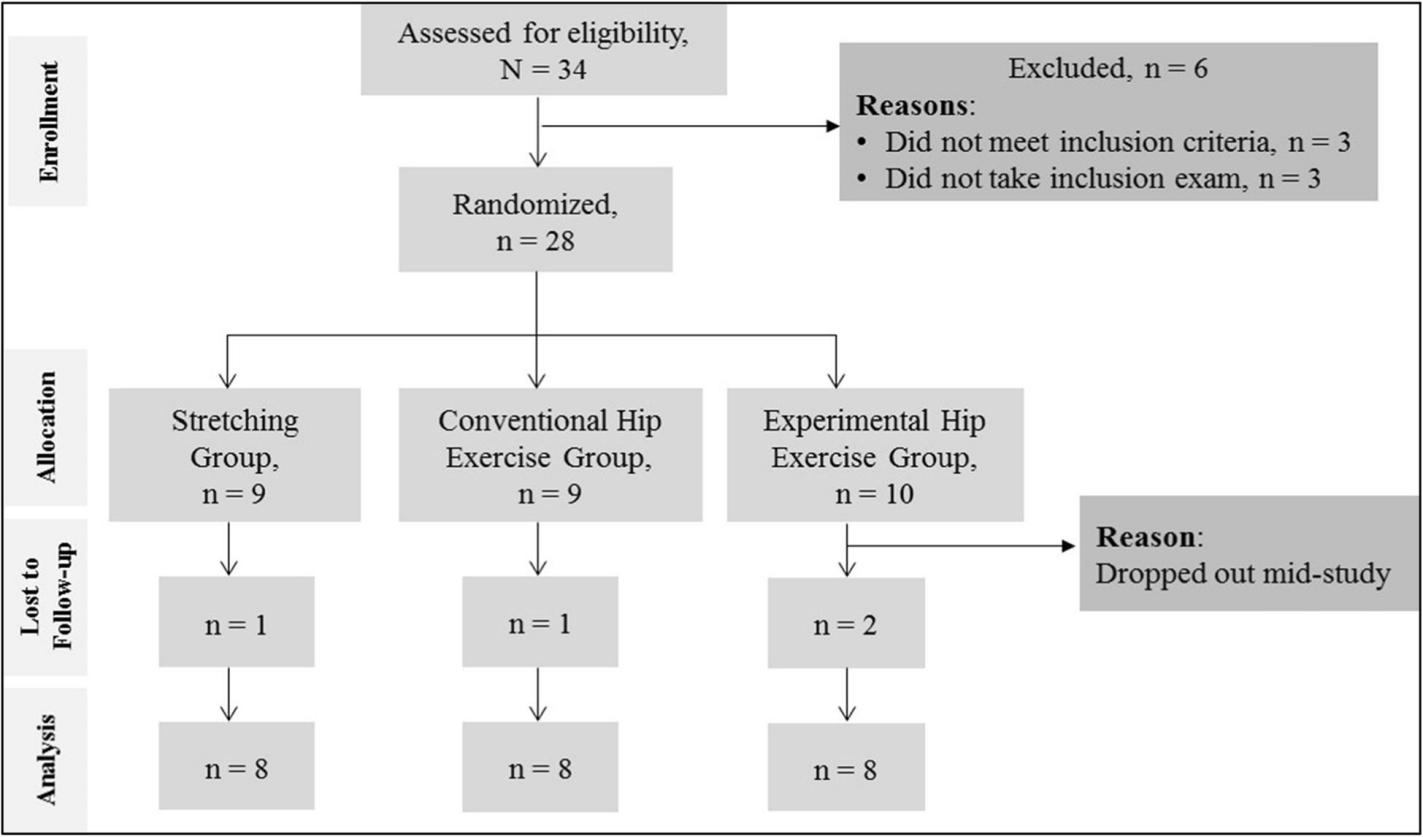
Study Flow and Participants Dispositions

Of the 34 screened female runners, 6 (16%) were not eligible (did not meet inclusion criteria) or chose not to participate in the study (did not take inclusion exam). Of the 28 patients who met the inclusion criteria, 4 participants dropped out mid-study. Thus, the study analysis included 24 participants who had been randomly allocated to three treatments groups (stretching group *n* = 8; conventional exercise group *n* = 8; and experimental exercise group *n* = 8). Study design and participant disposition is presented in Fig. 1. Baseline characteristics of participants in all three treatment groups are presented in the table submitted as additional electronic material.

In the stretching group, a statistically significant difference (*p* < 0.05) was observed for the YBT injured posterior lateral, SLMS injured knee-foot, SLMS injured knee-medial-foot, SLMS uninjured knee-foot, and SLMS uninjured knee-med-foot. The mean differences in outcome measures within groups are presented in the table submitted as additional electronic material. In the conventional exercise group, a statistically significant difference (*p* < 0.05) was observed for the YBT injured anterior, the uninjured DN, and the NPRS.

In the experimental group, a statistically significant difference (*p* < 0.05) was observed for the composite YBT uninjured and injured; the YBT for injured posterior medial and uninjured posterior lateral; the DN for uninjured and injured; the LEFS; the NPRS Run; and the SLMS for the injured knee-foot, injured knee-med-foot, uninjured knee-foot, and uninjured knee med-foot.

Between the conventional-stretching group, statistically significant differences (*p* < 0.05) were observed for the three parameters of YBT: injured anterior, injured posterior medial, and injured posterior lateral. Between the conventional-experimental group, statistically significant difference (*p* < 0.05) was observed only for the SLMS uninjured knee-foot. Between the experimental and stretching group, statistically significant differences (*p* < 0.05) were observed for the YBT uninjured anterior and injured anterior, the LEFS, and the SLMS uninjured knee-foot and uninjured knee-med foot.

Of three rehabilitation sessions per week over a span of 8 weeks, the mean and median for the number of rehabilitation sessions completed each week was 2.75 and 3 for the stretching group; 2.63 and 3 for the conventional group; 2.50 and 2.5 for the experimental group. None of our patients experienced a recurrence of symptoms within the timeframe of the study.

## Discussion

It was hypothesized that, by adding progressively increasing complexity of core exercises over an 8-week period, participants would experience less pain and improved function equal to or greater than the control groups (ITB stretching and conventional exercise). Baker and Fredericson found that, at 6 weeks, 22 of 24 runners were pain free and had returned to running, and suggested that 6 weeks should be the benchmark to consider whether a given treatment modality has been effective in those with ITBS [2]. A thorough search of the relevant literature yielded no studies comparing the effect of different exercise regimes among female runners.

The study results showed that neither 8 weeks of stretching of hip nor core strengthening significantly influenced the YBT™, DN muscle testing, LEFS, NPS, and SLMS indices, which may be attributed to the small sample size and short rehabilitation periods. However, all three exercise regimens produced improvements in clinical outcomes including pain, function, and strength; with the stretching and experimental group showing the greatest differences.

Within group comparison showed that, in the experimental group, a total of 13 outcome parameters showed significant differences. On the other hand, the stretching group and the conventional group showed significant differences for only five and three parameters, respectively. Thus, these results indicate that the experimental exercise regimen could be more beneficial for patients with ITBS than conventional exercises.

Traditional rehabilitation, which employed a mixture of isolated hip exercises, NSAIDs, icing, and reduction of aggravation has previously demonstrated to show improvement [27]. However, the effectiveness of these treatments varies according to the stages of ITBS during which each intervention manifested a maximum period of effectivenesss [12]. Thus, the findings of the study by Ellis et al. were consistent with the observations from this study which showed only moderate improvement in ITBS symptoms with conventional hip exercises [12].

Reduced gluteus medius strength leads to decreased stabilization and control [2]. The weakened gluteus medius muscle is known to be the cause of lower extremity dysfunction and injuries including ITBS [2, 28]. Several exercises activate the gluteus muscle, including single leg squat, clamshell, side-lying hip abduction [29, 30]. All the above-mentioned exercises were included in the experimental group of this study. Thus, these exercises in addition to the others would have led to the activation of gluteus medius, resulting in improved function and strength associated with ITBS.

Fredericson et al. also examined 24 distance runners (14 females) with ITBS treated with stretching and hip abduction exercises and pelvic drops (6 weeks rehabilitation program) [2]. Following the treatment, there was statistically significant (*p* < 0.05) increase in average hip abductor torque to 10.55% BWh, a 34.9% increase in injured female runners and to 10.38% BWh, a 51.4% increase in injured male runners. At the end of 6 weeks, 22 runners were pain free. and there was no recurrence of ITBS as per telephonic follow-up after 6 months [2]. Beers et al. (2008) examined the effectiveness of hip abductor strengthening in sixteen subjects (5 men and 11 women) aged between 20 and 53 years. A statistically significant difference (*p* = 0.05) in the strength was observed between the affected and unaffected limbs at baseline (week 0) which disappeared at the end of 6 weeks. The increase in hip abductor strength resulted in increased physical function capacity [18].

One of the major issue that hinders the effective treatment of ITBS is the fact that the cause of ITBS is difficult to identify [7]. Although there are standard protocols for treatment, the results vary. This could result from not recognizing the root cause of an individual’s ITBS, and thus not being able to address it adequately. Therefore, it is not surprising that the five indices used in this study to evaluate the treatment success were not sufficiently discriminating. Indeed, a thorough history of each patient should be collected including training age, gender, weight, previous injuries, training surface, training schedule, recovery schedule, proper tapering before competition, foot wear (i.e., examining shoe soles for uneven wear), running surface, and mileage accumulated over short or long time periods. Following this, a biomechanical assessment of any compensatory patterns or movement asymmetries due to previous injuries or inherent skeletal muscular mechanics should be performed. These total assessments lead to a functional diagnosis complementing the orthopedic diagnosis of ITBS. A functional diagnosis can either be tissue extensibility dysfunction, stability/motor control dysfunction, or joint mobility dysfunction and may include inflammation. Therefore, a customized regimen may be crafted including (1) rehabilitation to improve stability or muscle weakness, (2) joint manipulations/mobilizations to address joint dysfunction, (3) appropriate shoe adjustments (including potential orthoses), and (4) acupuncture, soft-tissue therapy, stretching and/or instrument assisted soft-tissue mobilization.

The experimental treatment group resulted in improvement in pain, function, and strength although the improvement was not statistically significant for some of the parameters assessed. The results of the study indicate that although the experimental treatment did not show statistically significant improvements in all the parameters assessed, it resulted in improvements in function, pain, and strength among study participants. These findings are valuable because they are a product of a body of research and present a new mode of treatment intervention. The study results emphasize that future research should be conducted with the experimental procedure to assess its effectiveness in large sample size with different weight, age, gender, and other variables.

Limitations of the present study include the small sample size and the short duration of the exercise intervention. With a high number of dropouts and exclusions, we acknowledge that we did not recruit all the subjects necessary to satisfy the ex ante power analysis. However, we were stringent in our recruitment selection, so as to only include in the study subjects who fully satisfied the inclusion criteria, and were likely to finish the study. Within the limits imposed by the number of subjects recruited, we are confident that our results are valid and representative. A larger sample is recommended to detect statistical differences between and within groups. A longer study period such as 10–12 weeks would allow time for myofascial adaptations to the exercises interventions. Some of the information for adherence and use of other treatments outside of study were self-reported by the subjects, which may have affected the accuracy of the treatment outcomes. Finally, this study did not account for fatigue and pain felt after a certain distance. Some of the runners did report that they did not feel pain until after 5 km. This may have affected the results of the NRPS outcomes and the return-to-run program given as well as the functional questionnaire. Further research is needed to compare the experimental exercise regimen and its effects on running biomechanics and fatigue. However, this was an exploratory study and based on the study findings a power calculation was performed and it was determined that a sample size of 12 patients for each treatment group was required for a power of 85%.We performed a power analysis based on a *t* test for each coefficient estimate of an ANOVA model. The statistics of the present investigation were undertaken using a per protocol analysis, since the dropouts were removed in the analysis. This study acknowledges that the intention to treat analysis is generally a more robust analysis, but emphasize that there was roughly an equal number of dropouts in each group, and the effect of removing them is not likely to change the interpretation of the results.

Although a preliminary power analysis showed that, for this pilot study, a total of 12 patients per each group would have sufficed, we are aware that such small numbers may engender a type I statistical error. However, given the nature of this preliminary investigation, the data produced should be used to inform more definitive studies.

## Conclusion

With the increasing popularity in running and the associated running-related injuries, there are increasing requirements to develop therapeutic strategies to treat these injuries. This study has attempted to draw together much of the evidence available in the literature to develop a new intervention program for the management of chronic ITBS. Previous research has shown that isolated hip exercises and stretching do have a positive effect on reducing the symptoms of ITBS [13, 19]. The results of this study demonstrate that the experimental exercise may be a good intervention based on the overall reduction in pain, and improvement in strength and function in individuals with chronic ITBS. Sports medicine practitioners and strength and conditioning coaches aim to reduce the risk of injury and provide prevention strategies to their patients and clients. This study has introduced a new interventional progressive strengthening program that can be utilized in the management of chronic ITBS to reduce pain and improve function.

## Data Availability

ClinicalTrials.gov identifier (NCT number): NCT0229615

## Abbreviations

ITBS: Iliotibial band syndrome
NPRS: Numeric pain rating scale
LEFS: Lower extremity functional scale
SLMS: Single-limb mini squat
YBT: Y-balance test™
DN: Dynamometer
ITB: Iliotibial band
LFC: Lateral femoral epicondyle
NSAIDs: Non-steroidal anti-inflammatory drugs
UBC-REB: Clinical Research Ethics Board of the University of British Columbia
NSCA: National Strength and Conditioning Association
HHD: Hand-held dynamometer
SEBT: Star excursion balance test
LQ: Lower quarter
SD: Standard deviation
